# Supported employment as a global mental health intervention

**DOI:** 10.1017/gmh.2024.112

**Published:** 2024-10-24

**Authors:** Franco Mascayano, Robert E. Drake

**Affiliations:** 1 Division of Behavioral Health Services and Policies, New York State Psychiatric Institute, New York, NY, USA; 2 Department of Epidemiology, Mailman School of Public Health, Columbia University, New York, NY, USA

## Abstract

The global health community has recognized that social determinants of health account for most of the inequities of health outcomes, including mental health outcomes, across and within countries. Strategies to overcome such inequities must focus on modifiable social factors. In this viewpoint, we argue for the preeminence of employment among social determinants of mental health for several reasons. People with mental health disabilities want to work, and a well-validated model of supported employment that is effective and cost-effective now exists. Employment leads to improvements in income, daily structure, self-esteem, social support, community integration and illness management, and people who are employed experience fewer emergencies and hospitalizations. Employment is empowering because people can use added income to activate their own choices regarding other social determinants. Supported employment actualizes the recovery paradigm: People who are employed in competitive jobs of their choice develop a meaningful functional life, increased self-esteem and new social supports. We provide examples of supported employment developments in diverse settings and discuss the implications of scaling up these services worldwide.

## Impact statement

This viewpoint highlights the critical role of employment as a modifiable social determinant of mental health. Supported employment, particularly through the Individual Placement and Support (IPS) model, has been demonstrated to improve employment outcomes and broader outcomes such as self-esteem, daily structure and community integration. This paper underscores the potential of scaling up IPS to transform mental health services worldwide, particularly in low- and middle-income countries. The impact of this work lies in its potential to guide policymakers, practitioners and global health advocates in addressing social inequities by integrating supported employment into mental health services, thereby promoting recovery and enhancing quality of life for people with mental health conditions.

## Introduction

Social determinants account for a large proportion of inequities in health and mental health outcomes (Lund et al., 2018). In this context, certain social factors are more modifiable than others. Modifiability refers to the extent to which interventions can effectively alter these factors to improve outcomes. Modifiable social factors include employment, education or training, and housing. This article aims to highlight the importance of supported employment as a critical intervention for addressing social determinants of health and its potential for global implementation. By examining the evidence base for supported employment, discussing its applications in different socioeconomic contexts and addressing the challenges and opportunities for its implementation, we aim to provide an overview of how supported employment can transform mental health services and outcomes globally. We discuss separate developments in the Global North, which we define as high-income, or Organization for Economic Cooperation and Development countries, and the Global South, which comprises low- and middle-income countries.

## Social determinants of health

Social determinants encompass conditions in which people are born, grow, live, work and age, including factors like socioeconomic status, education, physical environment, employment and social support networks (Lund, 2023). Access to health care and health behaviors such as obesity, sedentary lifestyle, alcohol abuse, drug use, tobacco use and family disruptions are key factors influenced by social determinants. Health care accounts for only 20% of health outcomes, while social determinants and related behaviors determine nearly all the rest (Hood et al., 2016).

Mental health is no different. International mental health researchers and organizations have increasingly emphasized the pernicious effects of social determinants (Allen et al., 2014; World Health Organization, 2014; Alegria et al., 2018; Patel et al., 2018). The United Nations has emphasized the need to confront the social, economic and political determinants of mental health through comprehensive social interventions (Puras, 2019). The World Health Organization (WHO, 2022a) and the Lancet Commission on Global Mental Health and Sustainable Development (Patel et al., 2018) have supported the need for macro-level social challenges to address inequalities in mental health outcomes.

The field of global mental health, which traditionally focused on expanding mental health services to underserved populations, has recently emphasized the importance of the social determinants of mental health (Lund et al., 2018; Lund, 2023). The 2018 Lancet Commission on Global Mental Health and Sustainable Development found that social determinants, such as income inequalities and gender-based violence, are important drivers of the mental health of populations, including those with a mental disorder (Lund et al., 2018). While expanding mental health services remains important, addressing social conditions could supersede traditional health care and transform lives (Kirkbride et al., 2024). As Michael Marmot stated nearly 20 years ago: “If the major determinants of health are social, so must be the remedies” (Marmot, 2005).

Global disability rights legislation, such as the United Nations Article 27, promotes opportunities for people with disabilities, including people with psychiatric disabilities (“Promote employment opportunities and career advancement for persons with disabilities in the labor market, as well as assistance in finding, obtaining, maintaining, and returning to employment”) (Anand, 2021). Moreover, the Sustainable Development Goals (SDGs) include a goal related to employment and career development (Goal 8: “to promote inclusive and sustainable economic growth, employment, and decent work for all”) (United Nations General Assembly, 2015). Individual countries have legislated equal opportunity for historically marginalized and currently minoritized groups. In 2016, a study on global work legislation reported that 114 UN members (59%) had laws promoting employment for individuals with mental health problems through different concrete actions (Nardodkar et al., 2016). In the US, the Americans with Disabilities Act (United States Congress, 1990) requires that state mental health authorities provide effective employment services to individuals with serious mental disorders. Furthermore, the human rights movement asserts that people with a disability should have the opportunity to work and earn money like other citizens (Johnson, 2005). To do otherwise relegates millions of individuals with mental health conditions to a marginal existence, termed the “disability gulag” (Johnson, The Disability Gulag, NY Times, Nov 23rd, 2003).

## Employment is a central social determinant of mental health

Addressing social determinants must target modifiable factors such as employment (Kamdar et al., 2023). We argue that employment is a central, modifiable social determinant of mental health for several reasons. First, most people with mental health disabilities want to work (Bond et al., 2012a; Bond and Drake, 2014; Wescott et al., 2015). They prefer competitive employment to additional government support because they want to be independent, functional, contributing citizens. Second, a well-validated model of supported employment – Individual Placement and Support (IPS) – that is effective and cost-effective has a well-established evidence base (Drake and Bond, 2023). Current research, including independent systematic reviews (e.g., Modini et al., 2016; Brinchmann et al., 2020), shows that many people with a mental disorder, even those with serious, disabling conditions, can succeed in competitive employment with the aid of supported employment (Bond et al., 2020; Knapp and Wong, 2020). IPS is the only supported employment intervention with repeatedly demonstrated outcomes across various studies and contexts. Third, empirical evidence shows that work conduces to health. For example, longitudinal studies have demonstrated that employment leads to improvements in not just income but also daily structure, self-esteem, social support, community integration and illness management (Rueda et al., 2012; Luciano et al., 2014), supporting the causation hypothesis. Historically, Hippocrates recognized the association between health and work around 400 BCE. Fourth, although IPS may not replace other costs and can be seen as an additional service, it has been shown to be cost-effective in the long term by reducing hospitalizations and improving overall outcomes (Knapp and Wong, 2020; Drake and Bond, 2023). Fifth, employment empowers individuals because they can use earned income to activate their own choices regarding other social needs (Bond et al., 2001). Finally, employment actualizes the recovery paradigm: People who are employed in jobs of their choice develop a meaningful functional life, friends outside of the mental health system and community integration (Deegan, 1988).

## Evidence-based supported employment and recovery

IPS-supported employment, backed by more than 30 randomized controlled trials, has emerged as a widespread mental health intervention (Drake and Bond, 2023). While most mental health interventions for schizophrenia, bipolar disorder and major depression prioritize diagnosis and symptom management, they have a limited impact on enhancing social functioning. In fact, traditional treatments often reduce symptoms without improving social functioning (Percudani et al., 2004; Harrington, 2019).

IPS-supported employment provides a simple, direct, model for assisting people with a mental disorder to obtain and sustain a competitive job. One of the key principles of IPS is integrating individualized vocational support with ongoing mental health care, ensuring that participants receive holistic support that addresses both their employment and mental health needs. This integration is facilitated through close collaboration between employment specialists, mental health professionals and other service providers, creating a coordinated support network that enhances the overall effectiveness of the intervention. At least two dozen high-income countries have embraced IPS as a critical mental health intervention (Drake and Wallach, 2020), meaning they recognize its value and potential. However, not all these countries have translated that ideology into public policy, meaning they have not fully integrated IPS principles into their national mental health policies and funding mechanisms (Drake et al., 2013). Many countries are in fact implementing and funding supported employment as a standard mental health intervention (Drake and Bond, 2023).

The evolution of IPS for people with mental disorders has followed established guidelines for evidence-based practices: stepwise development of an evidence-based intervention, strengthening the evidence base, identifying key elements, conducting independent systematic reviews, developing quality and outcome measures and implementing the intervention to improve outcomes broadly (Institute of Medicine, 2015). Publications by Drake and Bond (2023) and Bond et al. (2020) exemplify these steps, demonstrating that the process has been intentional and systematic. Evidence on efficacy, effectiveness, cost-effectiveness, systematic reviews and meta-analyses shows that evidence-based supported employment consistently increases competitive, integrated employment and that those who become employed benefit in economic, psychological and social domains (Frederick and VanderWeele, 2019; Brinchmann et al., 2020; Drake and Bond, 2023). Over half of participants in supported employment trials – two to three times more than those who receive other employment interventions – achieve employment success without harmful side effects (Frederick and VanderWeele, 2019; Brinchmann et al., 2020). While IPS has been shown to significantly improve employment outcomes, approximately 30–40% of participants do not achieve competitive employment, highlighting the need for additional examination of IPS effects among nonresponders. Some recent studies show that adding cognitive enhancement can improve employment outcomes for nonresponders (McGurk et al., 2015).

The IPS Employment Center has refined implementation procedures, including the use of an international learning community, over the past three decades (Bond et al., 2020). Moreover, researchers have developed quality measures that assess the key elements of supported employment through standardized, validated fidelity measures and outcome measures that assess several dimensions of employment (Bond et al., 2012a). Learning community procedures include education, data sharing and regular meetings for self-help among peers (Becker et al., 2014). Research shows that programs using learning community procedures achieve higher fidelity scores and are more likely to sustain and expand supported employment services (Pogue et al., 2022).

IPS-supported employment actualizes the current mental health recovery paradigm, which promotes a meaningful, functional life beyond symptom control (Deegan, 1988; Anthony, 1993; Hogan, 2002; Whitley and Drake, 2010; Slade and Longden, 2015). As America’s leading psychiatric advocate, Dr. Patricia Deegan has argued for decades, people with mental disorders view “recovery” as achieving a meaningful, active, functional life, not as a complete absence of symptoms (Deegan, 1988). Work can contribute significantly to an individual’s sense of purpose and self-worth, and people can learn to tolerate and cope with symptoms if they have a life that they consider valuable (Drake and Wallach, 2020).

While IPS is effective in achieving competitive employment, it must be complemented by policies that ensure meaningful and respectful work environments, as outlined in WHO (2022a) and OECD (2024) guidelines. For example, in Western Europe, the implementation of IPS has shown significant success due to strong policy support and funding mechanisms that facilitate the integration of employment services into the vocational rehabilitation and mental healthcare systems. In contrast, in the Global South, the sociopolitical landscape requires adaptations to the IPS model to account for the high levels of informal employment and the crucial role of family support in vocational rehabilitation. In the next sections, we present examples that highlight the importance of tailoring IPS implementations to fit the unique sociopolitical and cultural contexts of each region, which enhances the contextual fit and sustainability of supported employment interventions.

## IPS in the Global North

IPS services have spread rapidly in the Global North’s success. More than two dozen countries in North America, Europe and Asia are now implementing IPS programs. Most of this development has occurred in the past 10 years. We estimate that over 1,000 IPS programs exist in the US and another 1,000 in other countries (Drake and Bond, 2023). Programs have from one to a dozen or more IPS specialists, each of whom provides services to about 30 individuals per year. If we assume that a typical IPS program has two or three specialists, we estimate that 60,000 to 90,000 individuals receive services in the US each year and a similar number receive IPS in other countries.

In Northern Europe, countries such as Sweden, the Netherlands and the UK have integrated IPS into their national mental health services with considerable success. For instance, Sweden has implemented IPS in several municipalities, demonstrating a significant impact on employment outcomes for people with severe mental illness (Jónasson et al., 2022). Similarly, the Netherlands has integrated IPS into their existing vocational rehabilitation services, which has resulted in higher employment rates and improved quality of life for participants (Vukadin et al., 2024). In Southern Europe, countries like Italy and Spain have started to adopt IPS models with adaptations to fit their specific socioeconomic contexts, showcasing the flexibility and effectiveness of IPS in diverse settings. The European Learning Collaborative has been instrumental in sharing best practices and facilitating the adaptation of IPS across different European contexts (Jónasson et al., 2022). Twenty-two countries attended the most recent European IPS Learning Community meeting.

The goal in the Global North is scaling up services to reach more people with mental health disability (Drake and Bond, 2023). Therefore, future endeavors should aim to expand IPS services to new settings and populations, with a special emphasis on underrepresented groups, such as young individuals from minority backgrounds, immigrants and people experiencing homelessness. Crucial for IPS expansion is its integration into existing policies covering other social determinants of health such as housing, education and policy welfare. For instance, in the UK, IPS has been integrated into the broader framework of the National Health Service (NHS) and local government initiatives aimed at improving mental health and well-being through coordinated support across multiple sectors. By aligning IPS with housing support programs, educational initiatives and social services, the UK is trying to develop a more holistic approach to mental health recovery and social reintegration. Similarly, in the Netherlands, IPS has been incorporated into the national policy on disability and employment, ensuring that individuals with mental health conditions receive coordinated support that addresses their broader social determinants of health (Vukadin et al., 2024).

Scaling up IPS requires a nuanced understanding of each nation’s unique economic, social and policy landscapes, as well as specific barriers and facilitators. In the US, simplification of the payment system could play a pivotal role across states (Drake et al., 2013). Countries like Japan and South Korea, which have robust technological infrastructures but traditionally less emphasis on community-based services, may require a cultural shift toward such services. For Northern Europe, where comprehensive welfare systems provide significant support for unemployment, a recalibration could encourage and reward return-to-work efforts and prevent long-term dependency on government assistance (Knapp et al., 2013).

## IPS in the Global South

In the Global South, the recovery paradigm is not universally known, and IPS services are rarely available. Some emerging initiatives exist, but most people in need do not receive supported employment services. Although one could argue that introducing standard IPS is justifiable, we suggest a more careful and local approach. Before exploring the potential of supported employment, we should identify the unique values, barriers and opportunities in these contexts. Below, we use India, Mexico and South Africa as examples. We chose these three countries due to their diverse socioeconomic landscapes and emerging initiatives in supported employment for individuals with psychiatric disabilities, which provide a varied sample for discussing IPS implementation in the Global South.

In India, a pilot study illustrated the potential and hurdles of implementing IPS (Jagannathan et al., 2020). Out of 63 participants recruited into the study, 32 (50.8%) participants were placed in competitive jobs, underscoring the viability of the model in Indian settings. However, adapting IPS to India’s socioeconomic landscape requires navigating systemic constraints regarding access to vocational services and health care in general, as well as considering the role of families and communities (Khare et al., 2020; Khare et al., 2022). In this context, the adaptation of IPS must capitalize on the existing familial support structures, leveraging the family’s role in case management and employment facilitation. Moreover, the IPS model in India might need to be flexible, incorporating self-employment and informal job sectors that are vital in rural areas, where much of the population resides and where the formal job market is less prevalent (Sivakumar and Thirthalli, 2023).

In Mexico, an ongoing pilot RCT of standard IPS is being conducted in close collaboration with the national employment office (Saracco et al., 2023). This office provides employment services for people with or without a disability. This partnership could serve as a model for fidelity to IPS principles in the Global South, ensuring that the supported employment services align with local infrastructures and social services already in place. The Mexican experience suggests that, contrary to initial expectations, formal employment opportunities are accessible and can be utilized effectively for IPS services, even within a landscape where the informal job market plays a significant role.

South Africa, with its unique history and socioeconomic challenges, presents a different set of challenges for implementing IPS (Van Niekerk et al., 2015). The high unemployment rate in the general population and the legacy of social and racial inequalities may require adaptations to IPS such as fostering entrepreneurial skills and microenterprise development (Karanda and Toledano, 2012). This approach aligns with local self-employment models, which may be more feasible in areas with limited formal employment opportunities (Littlewood and Holt, 2018). Additionally, South Africa’s strong community-based organizations and social movements (Dawson and Sinwell, 2016) could be leveraged to provide the community support vital for the success of IPS programs.

Across these diverse contexts, a decolonial approach to IPS in the Global South implies that the standard model should be adapted, not just adopted (Bemme and Kirmayer, 2020). This approach requires a culturally sensitive application that respects local knowledge systems and care practices (Bhakuni and Abimbola, 2021). It means building upon local strengths, such as the tight-knit community networks in India, the governmental collaboration in Mexico and the potential for social entrepreneurship in South Africa. In particular, community-based rehabilitation models are widely used in the Global South and focus on empowering individuals with disabilities through community involvement and support. Integrating IPS into these models can leverage the strengths of both approaches, providing comprehensive support that addresses both vocational and community integration needs. For example, in rural areas of India, community-based programs that involve local communities and families in the rehabilitation process can be enhanced by incorporating IPS principles to provide structured employment support. This integration can create a more robust support system that addresses the unique challenges faced by individuals with mental health conditions in these settings. Such an approach would not only address the societal barriers and stigma but also work within the dynamics of the local job markets. Rigorous evaluation of these adapted IPS models is crucial, as it would contribute to a growing body of knowledge on culturally and contextually appropriate evidence-based practices. Such research would not only validate these adaptations but also guide the scaling up of IPS services in the Global South, ensuring that they are effective, valid and sustainable.

## Conclusions

Mental health disorders entail two main components: symptoms of illness and related social function difficulties. Treatment must therefore aim toward more than suppressing symptoms; addressing modifiable social factors, such as employment, is critical. The opportunity to pursue a meaningful life – central to the recovery paradigm – is a disability right and a fundamental human right. We can improve mental health care and functional outcomes by embracing supported employment as an essential mental health service. Employment can improve psychosocial functioning in general and can enable individuals to address other social determinants on their own terms and in their respective settings.

In addressing the challenges of scaling up evidence-based supported employment, we have identified several key issues such as the shortage of mental health professionals, lack of funding, insufficient legislative support and limited awareness and training about the IPS approach. To overcome these challenges, we propose a multifaceted strategy that includes capacity building, policy advocacy, task-shifting strategies and community engagement. For instance, increasing training programs for mental health professionals on IPS can help address the shortage of skilled practitioners. Additionally, advocating for policy changes that allocate more funding and resources toward supported employment and education can create a more sustainable framework for IPS implementation. Engaging communities and raising awareness about the benefits of supported employment can also foster a more supportive environment for individuals with mental health conditions.
