# Supplementary material for: Supported employment as a global mental health intervention
Source: Glob Ment Health (Camb). 2024 Oct 24;11:e102. doi: 10.1017/gmh.2024.112 (PMC11504922; doi:10.1017/gmh.2024.112)
Supplement: Peer review history

# Editor report: Recommendation: Supported employment as a global mental health intervention — R0/PR2

**DOI:** 10.1017/gmh.2024.112.pr2

**Affiliations:** Columbia University, United States

No accompanying comment.

# Editor report: Decision: Supported employment as a global mental health intervention — R0/PR3

**DOI:** 10.1017/gmh.2024.112.pr3

**Affiliations:** Johns Hopkins University Bloomberg School of Public Health, United States

No accompanying comment.

# Editor report: Recommendation: Supported employment as a global mental health intervention — R1/PR5

**DOI:** 10.1017/gmh.2024.112.pr5

**Affiliations:** Columbia University, United States

No accompanying comment.

# Editor report: Decision: Supported employment as a global mental health intervention — R1/PR6

**DOI:** 10.1017/gmh.2024.112.pr6

**Affiliations:** Johns Hopkins University Bloomberg School of Public Health, United States

No accompanying comment.
